# Interleukin-4 and its receptor alpha in paediatric uncomplicated malaria patients from a Ghanaian case-control study

**DOI:** 10.1186/s13104-024-06984-5

**Published:** 2024-10-31

**Authors:** Patrick Diaba-Nuhoho

**Affiliations:** 1 Department of Chemical Pathology, University of Ghana, Korle-Bu, Accra, Ghana; 2 Department of Paediatric and Adolescent Medicine, Paediatric Haematology and Oncology, University Hospital Münster, 48149 Münster, Germany

**Keywords:** Single nucleotide polymorphism, Malaria, Interleukin-4, Interleukin-4 receptor, Ghana

## Abstract

**Objectives:**

This study investigated gene polymorphisms in the interleukin-4 (IL-4) and its receptor alpha (IL-4Rα) gene regions in human hosts with uncomplicated malaria.

**Data description:**

Blood samples were obtained from a case-control study conducted at the Sogakope district hospital in the Volta region of Ghana. Thick blood films were made and used to detect the presence and levels of parasitaemia in the patient samples. Genotyping of IL-4 (150 C/T) and the IL-4Rα (Pro-478-Ser) polymorphisms in the promoter regions and receptor gene was performed using polymerase chain reaction-restriction fragment length polymorphism (PCR-RFLP) after DNA extraction. The data are useful in determining genetic polymorphisms (allele and genotypic frequencies) of IL-4 and its alpha receptor. In addition, they are useful when comparing levels of parasite density and haematological parameters between genotypic variants of IL-4 and IL-4Rα. These data contribute to our understanding of the genetic basis of malaria susceptibility, particularly in the population of the Volta region of Ghana.

## Objective

Malaria remains a major public health problem in Africa, it is hyperendemic presenting a serious problem in Ghana. *P. falciparum (Plasmodium falciparum)* is the predominant cause of severe morbidity and mortality in Ghana [1]. In children under 5 years of age, the incidence of Plasmodium parasites in the Volta region of Ghana was 20% [2]. Cytokines are important in immune mechanisms. Polymorphisms in cytokines affect immune response during infection with Plasmodium parasites, influencing susceptibility to various infectious diseases and affecting the balance between anti-inflammatory and pro-inflammatory cytokines [3]. IL-4 gene is located on chromosome 5q31-q33 [4] and blood infection levels of *Plasmodium falciparum* are linked to this chromosome region [5, 6]. In this region, candidate genes encode cytokines, growth factors and their receptors that are involved in the control of immunity during malaria infection [7]. In human *Plasmodium falciparum*, IL-4 is shown to be involved in the regulation of activated antibody response [8, 9]. In Sudanese children infected with *Plasmodium falciparum*, serum levels of IL-4 correlated with hyperparasitaemia rather than the clinical severity of the disease [10]. Absence of IL-4 in mice are protected from cerebral malaria [11] and mice treated with IL-4 analogous were protected against development of cerebral malaria by decreasing parasitaemia levels, inflammation and decreasing levels of cytotoxicity of T-cells [12]. Since genetic polymorphisms is known to contribute to susceptibility, severity of infectious autoimmune diseases and other complex diseases [13], this study aimed to address this gap in knowledge by investigating the involvement of gene polymorphisms in interleukin-4 (IL-4) and its receptor alpha (IL-4Rα) gene regions in uncomplicated malaria (UM) in paediatric Ghanaian patients. Unfortunately, these data were not published since the experiment was completed several years ago due to limited resources. The data presented here may be useful in future project planning related to this topic.

### Data description

This data set consists of a case-control hospital-based study conducted at the department of child health, Sogakope district teaching hospital in the Volta region of Ghana, the ethnic group were predominately the Ewe tribe. The participants were chosen from among 200 children with UM between the ages of 6 months and 12 years who were receiving medical care. The inclusion criteria were fever (> 37.5 °C) measured within 24 h of admission and malaria parasitaemia. In addition, the patients were sickling negative and did not have any other disease. UM cases were defined as patients with malaria parasitaemia > 2500 parasites/µl and haemoglobin level > 8 g/dl, with no other complications and full consciousness. The controls were children of the same age range as those who presented to the same hospital with relatively mild illness requiring outpatient treatment but who were negative for Plasmodium parasites. The data were collected using an interviewer-administered questionnaire (Data file 5, Table 1). Venous blood samples from patients with UM and controls were collected in sterile EDTA tubes before admission. The buffy coats were collected and stored at -20 °C until use. Thick blood smears were stained using 5% Giemsa solution and examined for Plasmodium species by two laboratory technicians. Parasite densities were determined by calculating the number of P. falciparum parasites per ml of blood. Genomic DNA was isolated from buffy coat samples using a Qiagen DNA extraction kit (Qiagen Co., UK) following the manufacturer’s protocol (Data file 4, Table 1) and stored at -20 °C. When single nucleotide polymorphisms (SNPs) in IL-4 and IL4-Rα were detected via polymerase chain reaction-restriction fragment length polymorphism (PCR-RFLP), the IL-4 promoter polymorphism (-590 C/T) was analysed via specific PCR amplification and BsmFI restriction endonuclease digestion (New England Biolabs Inc., USA). IL4-Rα polymorphisms were genotyped using PCR-RFLP analysis with PCR products digested with the restriction enzyme KpnI (New England Biolabs Inc., USA) under conditions described previously by Walley, Cookson [14]. PCR amplification and the RFLP products were analyzed using ethidium bromide stained 2% and 4% agarose gel electrophoresis, respectively. The study was approved by the ethics and protocol review committee of the School of Allied Health Sciences, College of Health Sciences, University of Ghana. Study participants were adequately informed of the purpose, procedures and risks of the study. All participants signed informed consent forms (Data file 5, Table 1). Table 1 shows the demographic profiles of the study participants which included 49 patients (Data file 1) and 46 controls (Data file 2). The clinical profiles of study subjects which included parasite density, haemoglobin levels, total white blood cell count, neutrophil, lymphocyte, red blood cell and platelet count are indicated in Table 1 as data file 1 and data file 2. The allele and genotype frequencies of the IL-4–590 C/T and IL-4 r alpha Pro-478-Ser polymorphisms are shown in Table 1 as data file 1 and data file 2. Images of the PCR and restriction enzyme digestion results are shown in Data file 3 in Table 1. The data provided here provide useful information for estimating the effects of IL-4 and its receptor alpha on clinical outcomes, such as haematological variables and parasitaemia in paediatric UM in Ghana. Table 1, Data file 4 contains a complete description of the methodology.

**Table 1:** Overview of data set

Label	Name of data file/data set	File types (file extension)	Data repository and identifier (DOI or accession number)
Data file 1	Case Data	MS Excel file (.xlsx)	Harvard Dataverse (10.7910/DVN/VY6SVX) [15]
Data file 2	Control Data	MS Excel file (.xlsx)	Harvard Dataverse (10.7910/DVN/VY6SVX) [15]
Data file 3	Images of PCR and restriction enzyme digest	Images in MS Excel file (.xlsx)	Harvard Dataverse (10.7910/DVN/VY6SVX) [15]
Data file 4	Methodology	MS Word file (.docx)	Harvard Dataverse (10.7910/DVN/VY6SVX) [15]
Data file 5	Consent and questionnaire forms	MS Word file (.docx)	Harvard Dataverse (10.7910/DVN/VY6SVX) [15]

### Limitations

Seum levels of IL-4 and its alpha receptor were not measured. This made it impossible to ascertain the effect of the polymorphism on the production of IL-4 and its alpha receptor. In addition, not all the samples could be genotyped due to resource constraints. The genotype distribution of Pro478Ser polymorphism was not in agreement with the HW equilibrium which could be addressed with more adequate sampling size.

## Data Availability

The data described in this data note can be freely and openly accessed on Harvard Dataverse under (https://doi.org/10.7910/DVN/VY6SVX) [15]. Please see Table 1 for details and links to the data.

## Abbreviations

DNA: Deoxyribonucleic acid
EDTA: Ethylenediamine tetra acetic acid
PCR: Polymerase chain reaction
P. falciparum: Plasmodium falciparum
RFLP: Restriction fragment length polymorphism
SNP: Single nucleotide polymorphism
UM: Uncomplicated malaria
